# Assessing the influence of medications with antagonistic effects on low-dose oral minoxidil in patients with alopecia: A retrospective study

**DOI:** 10.1016/j.jdin.2024.05.010

**Published:** 2024-07-14

**Authors:** Deesha Desai, Ambika Nohria, Michelle Sikora, Michael Buontempo, Jerry Shapiro, Avrom S. Caplan, Michael Garshick, Kristen I. Lo Sicco

**Affiliations:** a University of Pittsburgh School of Medicine, Pittsburgh, Pennsylvania; b The Ronald O. Perelman Department of Dermatology, NYU Grossman School of Medicine, New York, New York; c New York Medical College, Valhalla, New York; d Hackensack Meridian School of Medicine, Nutley, New Jersey; e Leon H. Charney Division of Cardiology, New York University Grossman School of Medicine, New York, New York

**Keywords:** alopecia, antagonist, efficacy, hair loss, minoxidil

*To the Editor:* The off-label utilization of low-dose oral minoxidil (LDOM) has transformed the therapeutic landscape of alopecia, a condition with previously limited therapies.^1^ At higher doses, minoxidil functions as an antihypertensive agent, inducing relaxation of smooth muscle vasculature and vasodilation by influencing potassium channels.^2^ When administered at low doses for alopecia, it is currently presumed to augment microcirculation surrounding the hair follicle, stimulating hair growth, with minimal blood pressure (BP) impacts.^3^

Various prescription and over-the-counter medications may disrupt vasodilation and limit BP drops. These medications, which act in an “antagonistic” fashion to the MOA of LDOM’s antihypertensive properties, could potentially reduce its efficacy.^4^ This study aims to quantitatively assess the effect of these medications on LDOM treatment outcomes using trichometric hair measurements with HairMatrix (Canfield).

A New York University Institutional Review Board-approved retrospective analysis was conducted of patients seen by New York University Dermatology from January 1, 2009 to August 28, 2023 and prescribed LDOM.

The patient cohort included 71 patients (60.6% females, median age 37.29), most commonly diagnosed with nonscarring alopecia (91.5%) (Table I). Non-steroidal anti-inflammatory drug and stimulants were the most frequent antagonistic medications (Supplementary Table I, available via Mendeley at https://data.mendeley.com/datasets/t6cb3bmcgx/1).

**Table I:** Patient demographics

	Overall cohort (*n* = 71)	No use of “antagonistic medications” (*n* = 52)	Use of “antagonistic medications” (*n* = 19)
Median age in years (range)	37.29 (16-84)	37.5 (16-78)	36.7 (18-84)
Gender	Males: 28 (39.4%)	Males: 20 (38.5%)	Males: 8 (42.1%)
	Females: 43 (60.6%)	Females: 32 (61.5%)	Females: 11 (57.9%)
Alopecia subtype	Androgenetic alopecia: 51 (71.8%)	Androgenetic alopecia: 40 (76.9%)	Androgenetic alopecia: 11 (57.9%)
	Telogen effluvium: 2 (2.8%)	Telogen effluvium: 1 (1.9%)	Telogen effluvium: 1 (5.3%)
	Frontal fibrosing alopecia: 2 (2.8%)	Frontal fibrosing alopecia: 1 (1.9%)	Frontal fibrosing alopecia: 1 (5.3%)
	Androgenetic alopecia + telogen effluvium: 12 (16.9%)	Androgenetic alopecia + Telogen effluvium: 9 (17.3%)	Androgenetic alopecia + Telogen effluvium: 3 (15.8%)
	Androgenetic alopecia + Lichen planopilaris: 4 (5.6%)	Androgenetic alopecia + Lichen planopilaris: 1 (1.9%)	Androgenetic alopecia + Lichen planopilaris: 3 (15.8%)
Starting median minoxidil dose in mg (range)	1.25 (0.625-2.5)	1.25 (0.625-2.5)	1.25 (0.625-2.5)

The change in median trichometric width and density for the overall cohort was 2.94 μm and 21.06 hairs/cm^2^, respectively. For patients concurrently using antagonistic medications, the change in median trichometric width and density was 5.5 μm and 18.5 hairs/cm^2^, respectively. For patients not concurrently using antagonistic medications, the change in median trichometric width and density was 2.0 μm and 22.0 hairs/cm^2^, respectively. There was no significant difference in the change of trichometric width or density between cohorts (*P* = .337, *P* = .229 respectively). Regarding tolerability, 23 patients reported side effects with no significant difference in side effect incidence between cohorts (*P* = .27) (Table II).

**Table II:** Change in trichometric measurements and reports of side effects from initial visit to last documented follow-up

	Overall cohort	No use of “antagonistic medications”	Use of “antagonistic medications”	*P* value
Change in median trichometric width	2.94 μm	2.0 μm	5.5 μm	.337
	IQR = 12.0∗	±9.60†	IQR = 8∗	
Change in median trichometric density	21.06 hairs/cm^2^	22.0 hairs/cm^2^	18.5 hairs/cm^2^	.229
	±25.20†	±24.59†	±25.22†	
Reported side effects	Total: 23 (32.4%)	Total: 16 (69.6%)	Total: 7 (30.4%)	N/A
	Dizziness: 3 (4.2%)	Dizziness: 3 (18.8%)	Dizziness: 0 (0%)	
	Headache: 3 (4.2%)	Headache: 2 (1.3%)	Headache: 1 (14.3%)	
	Fluid retention/Edema: 1 (1.4%)	Fluid retention/Edema: 0 (0%)	Fluid retention/Edema: 1 (14.3%)	
	Hypertrichosis: 16 (22.5%)	Hypertrichosis: 11 (68.8%)	Hypertrichosis: 5 (71.4%)	

∗ Nonparametric - Interquartile range (IQR) 50%.

† Parametric - Standard deviation.

Our findings did not reveal a significant impact of antagonistic medications on LDOM alopecia treatment outcomes. Patients demonstrated increases in trichometric width and density, irrespective of antagonistic medications. These results align with prior research demonstrating equivalent efficacy of LDOM in patients concurrently undergoing treatment with antagonistic medications.^5^

Alopecia patients frequently present with comorbid conditions, necessitating careful consideration when formulating treatment plans. This is particularly true when utilizing LDOM for the treatment of androgenetic alopecia, as patients typically present in the third or fourth decade of life with a higher likelihood of concomitant medication use to manage comorbidities. Our findings underscore that LDOM remains effective in treating alopecia, even in medically complex patients with multidrug regimens involving antagonistic medications.

Our results also reaffirm the favorable side effect profile of LDOM and demonstrate that patients concurrently using multiple medications can still tolerate LDOM effectively.^5^

Retrospective design, sample size, and patient-reported side effects. BP changes were not recorded; however, previous studies have noted nonsignificant BP impacts.^5^

This study reveals that treatment response to LDOM for alopecia may not be affected by mechanistically antagonistic medications, reinforcing the efficacy and tolerability of LDOM. Providers should encourage patients to adhere to their concurrent medications without fear of impeding progress in their hair growth journey.

## Conflicts of interest

None disclosed.
